# A Simple GC-MS/MS Method for Determination of Smoke Taint-Related Volatile Phenols in Grapes

**DOI:** 10.3390/metabo10070294

**Published:** 2020-07-17

**Authors:** Zhiqian Liu, Vilnis Ezernieks, Priyanka Reddy, Aaron Elkins, Christian Krill, Kieran Murphy, Simone Rochfort, German Spangenberg

**Affiliations:** 1 Agriculture Victoria Research, AgriBio, 5 Ring Road, Bundoora, VIC 3083, Australia; Vilnis.Ezernieks@agriculture.vic.gov.au (V.E.); priyanka.reddy@agriculture.vic.gov.au (P.R.); Aaron.Elkins@agriculture.vic.gov.au (A.E.); christian.krill@agriculture.vic.gov.au (C.K.); Kieran.Murphy@agriculture.vic.gov.au (K.M.); simone.rochfort@agriculture.vic.gov.au (S.R.); German.Spangenberg@ecodev.vic.gov.au (G.S.); 2 School of Applied Systems Biology, La Trobe University, Bundoora, VIC 3083, Australia

**Keywords:** grapes, volatile phenols, smoke taint, GC-MS

## Abstract

Volatile phenols (VPs) derived from smoke-exposed grapes are known to confer a smoky flavor to wine. Current methods for determination of VPs in grape berries either involve complex sample purification/derivatization steps or employ two analytical platforms for free and bound VP fractions. We report here a simple gas chromatography-tandem mass spectrometry (GC-MS/MS) method for quantification of both free and bound VPs in grapes, based on optimized (1) GC-MS/MS parameters, (2) an analyte extraction procedure, and (3) phenol glycoside hydrolysis conditions. Requiring neither sample cleanup nor a derivatization step, this method is sensitive (LOD ≤ 1 ng/g berries) and reproducible (RSD < 12% for repeated analyses) and is expected to significantly reduce the sample turnover time for smoke taint detection in vineyards.

## 1. Introduction

Wildfires and controlled burns generate smoke particulates that could be taken up by the berries and leaves of grapes in nearby vineyards. Wines made from smoke-exposed grapes can present undesirable sensory attributes known as smoky or smoke tainted with reduced palatability and market acceptance [1]. While the chemical composition of smoke from wood pyrolysis is rather complex, volatile phenols (VPs) are believed to be the major aroma compounds that confer the smoke taint characteristics to grapes and wines [1,2,3].

Guaiacol and 4-methylguaiacol were the first recognized VPs in relation to smoke taint [1,4]. Subsequently, other compounds especially cresols and syringol, were also found to be important contributors. As a result, seven VPs, including guaiacol, 4-methylguaiacol, m-cresol, p-cresol, o-cresol, syringol, and 4-methylsyringol, are currently monitored in analytical laboratories as markers for smoke taint appraisal in grapes and wines [3]. In addition, VP glycosides can be formed through conjugation with sugars once inside berry tissues and these non-volatile compounds are precursors from which free VPs could be released during vinification or wine storage [4,5]. Consequently, measuring glycosidically conjugated VPs is also essential for estimating the potential risk of producing smoke-tainted wines. 

Bush fires are expected to become more frequent in the coming years with global warming [1]. In order to minimize the financial loss associated with producing smoke-tainted wines, obtaining information on the level of free and bound VPs in grapes in the pre- or post-harvest stage in a timely manner is of critical importance. Currently, gas chromatography-mass spectrometry (GC-MS) and liquid chromatography-mass spectrometry (LC-MS) are the main analytical tools used for VP analysis. For free VP quantification, although LC-MS was used in rare cases [6], GC-MS has been the predominant analytical technique in combination with various sample preparation protocols, including liquid-liquid extraction (LLE) from berry homogenate and wine [7], headspace solid-phase microextraction (HP-SPME) from beer [8], polymer stir bar sorptive extraction from beer and wine [9], and trimethylsilyl (TMS)-based derivatization following purification by solid-phase extraction (SPE) of berry homogenate and wine [10]). 

In the case of glycosidically bound VPs, direct analysis has been reported only with LC-MS and in most studies following an SPE cleanup. In addition, due to the lack of authentic VP-glycoside standards, a single labelled internal standard was often used to estimate all VP-glycoside species [11,12]. Given the large number of potential VP glycosides in grapes [5], accurate quantification of even the major species is difficult to achieve. As a result, indirect quantification of the major aglycones after hydrolysis is a simpler option and such an approach was already attempted in several reports [13,14,15,16,17].

Currently, there is neither a consensus method concerning sample preparation for GC-MS analysis of free VPs in grape/wine samples, nor a standardized protocol for hydrolysis of VP-glycosides. The aim of this study was to develop and validate a simple GC-MS/MS method for both free and bound VP determination in grapes with optimized extraction and hydrolysis procedures. Moreover, nine additional VPs, including phenol, 4-ethylguaiacol, 4-ethylphenol, 2,4-dimethylphenol, 4-*n*-propylphenol, eugenol, isoeugenol, vanillin, and acetovanillone, that may also contribute to the smoke-tainted flavor of wine were included in this method. 

## 2. Results

### 2.1. Optimization of GC-MS/MS Method

**Figure 1:**
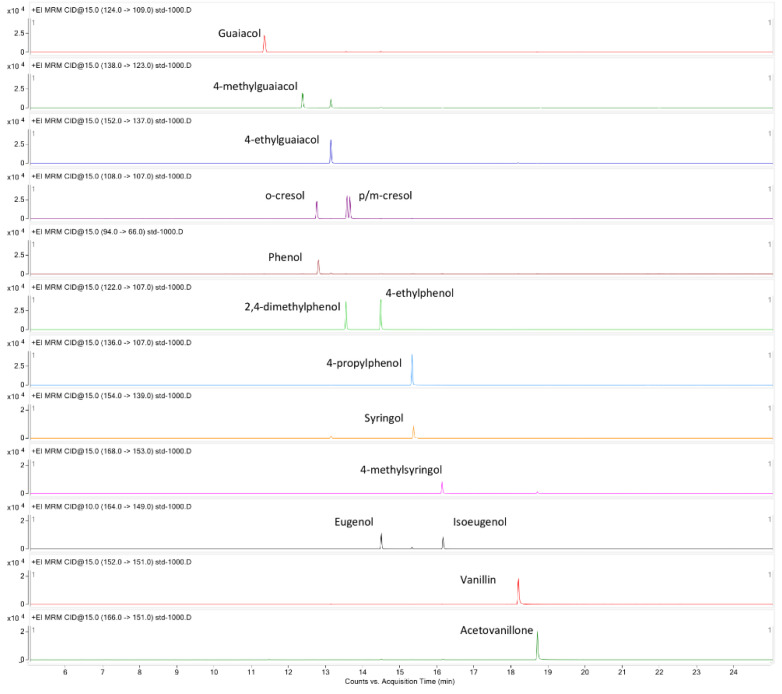
GC-MS/MS profile of 16 phenolic standards acquired in MRM mode.

By combining the DB-heavy-wax column separation with multiple reaction monitoring (MRM) scan mode, all 16 phenolic compounds, including 3 groups/pairs of isomers (*o/p/m*-cresol, eugenol/isoeugenol, and 2,4-dimethylphenol/4-ethylphenol) were resolved with a satisfactory peak shape in a total run of 25 min. Figure 1 shows the GC-MS/MS profile of the standards in MRM mode (at 1000 ng/mL). In addition, the high specificity of the MRM scan allowed a limit of detection (LOD) ≤ 1 ng/mL and limit of quantification (LOQ) between 1 and 3.3 ng/mL to be achieved for all 16 VPs. The LOD and LOQ values, linear range, and *R*^2^ for the 16 phenolic compounds under the current analytical conditions are summarized in Table 1. 

### 2.2. Selecting the Optimal Protocol for Extracting Free and Conjugated Phenols

**Figure 2:**
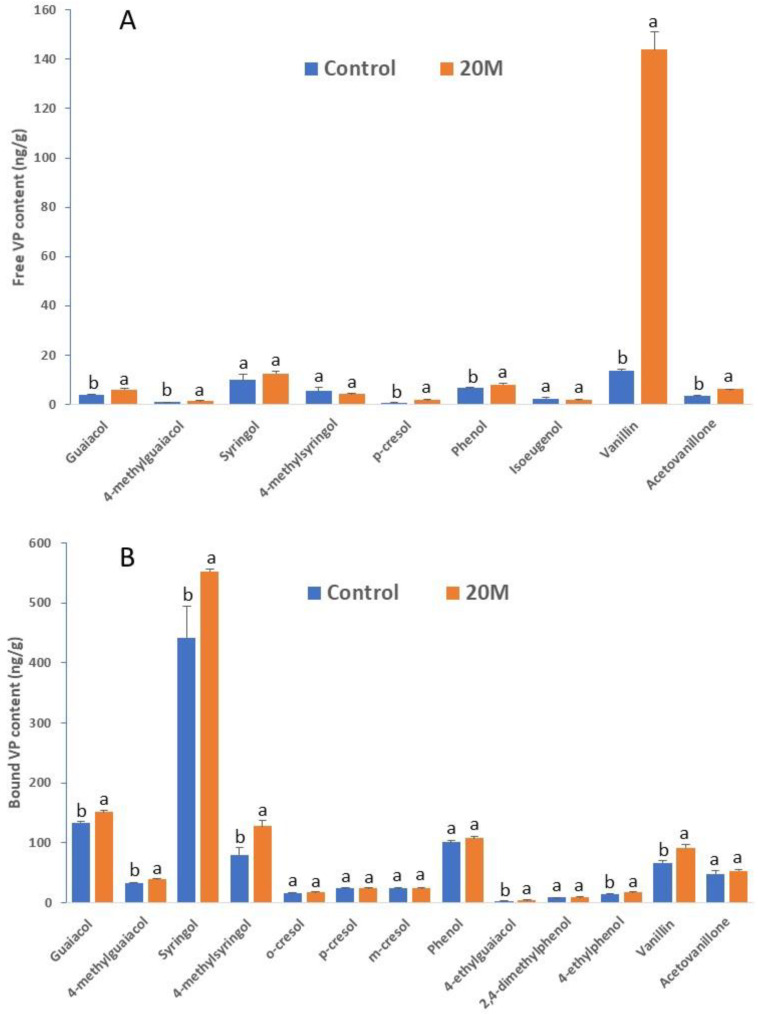
Content of free (**A**) and bound (**B**) VPs in a smoke-exposed grape sample (Shiraz) following different extraction protocols. Control: extraction with water at room temperature; 20M: extraction with 20% methanol at 80 °C (60 min). Columns represent the mean values of three replicates and error bars are standard deviations; within each compound, columns with different letters are significantly different (*p* < 0.05). Hydrolysis was conducted with 1.25 N H_2_SO_4_ for bound VP determination.

Compared with extraction by adding 40% of water (*v*/*w*, 4 mL to 10 g fruit homogenate) at room temperature (defined as protocol 1 or the control method in this report), extraction by adding 20% methanol (*v*/*w*, 2 mL to 10 g homogenate) combined with 60 min of heating (at 80 °C) (named protocol 2 or the 20M method in this report) yielded a higher content for six out of the nine free VPs detected in a sample exposed to a high-level of bush fire smoke; the biggest difference was observed with vanillin, for which a greater than 10-fold increase was observed as compared to the control method (Figure 2A). In addition, the 20M method showed an improved efficiency in extracting conjugated VPs; a higher content was observed for 7 of the 13 bound phenolic compounds detected in this sample (Figure 2B).

Indeed, when standards of VPs were spiked into the homogenate of non-smoked grape berries (at a level of 100 ng per g homogenate), a much higher recovery was obtained with the 20M extraction method as compared to the control method for the majority of the 16 compounds (Table 2). It is worth pointing out that even with the 20M extraction protocol, the recovery for most VPs was below 80% and two VPs 4-ethylphenol and 4-*n*-propylphenol even showed a recovery of less than 60%. 

### 2.3. Selecting the Optimal Conditions for Phenol Glycoside Hydrolysis 

**Figure 3:**
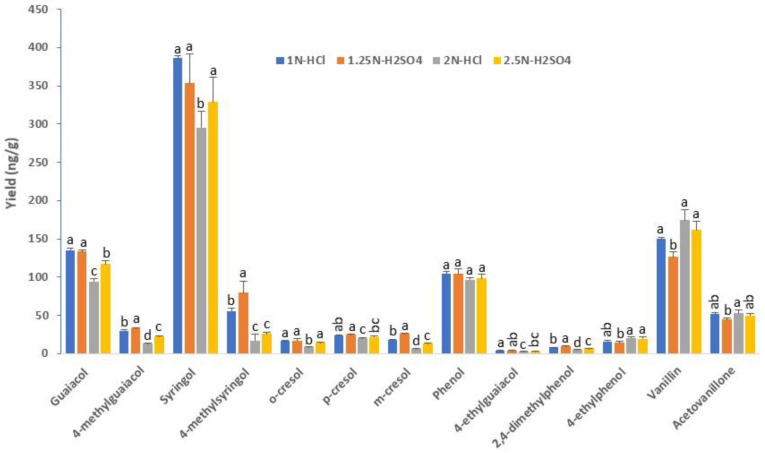
VP yield comparison following hydrolysis of a smoke-exposed grape sample (Shiraz) with different acids. Columns represent the mean values of three replicates and error bars are standard deviations. Within each compound, columns with different letters are significantly different (*p* < 0.05). All hydrolysis reactions were conducted at 100 °C for 60 min.

Four acidic hydrolysis procedures (1 N HCl, 2 N HCl, 1.25 N H_2_SO_4_, and 2.5 N H_2_SO_4,_ all combined with a 60-min incubation at 100 °C) were compared in this work using the 20M extract of a sample exposed to a high level of bush fire smoke. In total, 13 out of the 16 VPs were found after hydrolysis of this sample. Overall, a higher yield was obtained with 1 N HCl and 1.25 N H_2_SO_4_, whereas 2 N HCl and 2.5 N H_2_SO_4_ appeared to show a better performance only for vanillin (Figure 3).

The profile of guaiacol glucoside and two putative phenol diglycosides (guaiacol gentiobioside and pentose-hexose-cresol) before and after hydrolysis with 1 N HCl and 1.25 N H_2_SO_4_ was checked by LC-Orbitrap MS. While all the three compounds were present in the berry extract before hydrolysis, none of them were detectable after hydrolysis with 1 N HCl or 1.25 N H_2_SO_4_ (Supplementary Figure S1), indicating the high efficiency of these acids (and associated hydrolysis conditions) in cleaving phenol glycosides present in grape berry extract.

When VP standards were spiked (at 100 ng/mL) into the hydrolysis medium (containing 1.25 N H_2_SO_4_), recovery was 78–93% for most VP compounds, including the seven most important smoke taint-related ones (guaiacol, 4-methylguaiacol, syringol, 4-methylsyringol, o-cresol, p-cresol, and m-cresol) (Supplementary Table S1), indicating most of the VP compounds are tolerant to the hydrolysis conditions used in this study. By contrast, a very low recovery (<20%) was observed for isoeugenol. 

### 2.4. Method Accuracy

To verify the reliability of our method, we conducted an inter-laboratory comparison involving three commercial service laboratories, using sub-samples (Pinot Noir) collected from the same location. Table 3 shows that although the absolute values (both free and bound VP contents) are somewhat different across these four laboratories, the trends are all similar, noting that only seven VP compounds are routinely monitored in the commercial service laboratories for smoke taint analysis.

### 2.5. Method Reproducibility

The analytical precision of the method was assessed by parallel analysis of the same sample for five times for three grape cultivars Shiraz, Chardonnay, and Pinot Grigio (all exposed to a high level of bush fire smoke). Table 4 shows that both free and bound VPs vary substantially across the three cultivars, but the RSD of repeated analyses of the same sample was below 12% for most measurements across all cultivars, noting again that not all the 16 phenolic compounds were detected in these samples. This suggests that our method displays satisfactory reproducibility for determination of both free and conjugated VPs in grapes. 

### 2.6. Method Application

The optimized extraction and hydrolysis methods were applied to three types of grape samples (Shiraz) exposed to low, medium, and high levels of bush fire smoke (as scored visually by grape growers). For each sample type, three sub-samples (replicates) of 10 g of berries were randomly picked from different bunches and ground and extracted separately, and then the free and bound VPs were determined (Table 5). 

For free VPs, the content varies with individual phenolic compounds and also varies with sample types. In the case of the bound VPs, a higher number of species were detected as compared to the free VPs in these samples; the concentration of each VP is also higher in the bound form in most cases (except vanillin). Consequently, the total of bound VPs is far greater than that of the free VPs for each sample type. It should be noted that the sum of free and bound VPs matches the level of smoke exposure of the three types of samples. 

It is worth mentioning that for all three types of samples, the RSD is below 20% across the three replicates (sub-samples) for the majority of the measurements, indicating the method is suitable for quantifying both free and bound VPs in grape berries exposed to different levels of smoke.

## 3. Discussion

Developing a simple analytical method enabling a timely delivery of information regarding the VP content of smoke-exposed grapes is of increasing importance to grape growers and wine-making industries. GC-MS is known to be suitable for VP analysis and has been used for measuring smoke taint-related VPs in wine, beer, and grapes [2,8,9,10,17,18,19,20,21]. Through optimization of GC-MS/MS parameters, we were able to achieve an LOD around 1 ng/mL for all VPs, which is appropriate to estimate the level of VPs in grapes, which could potentially lead to smoke taint flavor of wine, given that the sensory limit for smoke taint of wine was between 20 and 100 ng/mL for the most studied VPs guaiacol and 4-methylguaiacol [4].

Regarding sample preparation for GC-MS analysis, Allen et al. [10] reported a fully validated method for phenolic compound analysis in grapes, but the method is rather complex because of the extra SPE purification and TMS derivatization steps. A rather simple LLE approach was adopted in several studies to transfer VPs from the supernatant of neat berry homogenate or wines to the organic phase amenable for direct GC-MS analysis [14,17,18]. This is by far the easiest sample preparation protocol for VP analysis by GC. However, while the LLE protocol was systematically evaluated previously [17], the first step of extraction (i.e., extracting VPs from grape berry homogenate) remained to be optimized. 

Given that aqueous methanol or ethanol is generally used for metabolite extraction in plant metabolic analysis [22], we tried various extraction regimes by varying the amount of methanol added and the incubation temperatures; a combination of adding 20% methanol (2 mL into 10 g homogenate) with 60 min of incubation at 80 °C was found to give the best overall yield for both free and bound VPs. Indeed, the extraction of phenolic compounds from grapes by adding methanol to the homogenate was described by Allen et al. [10]. We found that 50% methanol is more efficient for extracting free phenolic compounds, but the presence of a large proportion of methanol hinders the following LLE step; whereas the presence of up to 20% methanol in the extract matrix has no adverse effect on LLE, as evidenced by the near complete recovery (around 90%) of the majority of the spiked VP standards (data not shown). 

When VP standards were spiked into the berry homogenate, a relatively low recovery (70–85%) was observed for most VPs. This can be explained by the high ratio of fruit tissues (10 g) to the liquid in the extraction medium. A recovery of 48-66% was also reported by Noestheden et al. [17] for cresols and syringol. Indeed, a second extraction of the residues with 5 mL of 20% methanol could increase the recovery by 10–15%. Clearly, a single extraction is simpler but could underestimate (up to 10–15%) the level of free VPs; whereas two extractions will inevitably increase the sample processing time and reduce the throughput. Some VPs, such as 4-ethylphenol and 4-*n*-propylphenol, showed a rather low recovery (<60%) even with the 20M extraction method; the underlying cause remains to be investigated. Allen et al. [10] reported a satisfactory recovery (80–110%) of all VPs spiked into Merlot wine, but data is scarce in relation to the recovery of VPs from berry homogenate [17].

Smoke taint-related VP-glycosides in grapes and wines have been analyzed by LC-MS in several reports [5,7,11,12,14,20,23]. Due to the lack of authentic standards and the large number of VP-glycoside species in grapes [5,20], accurately measuring even the major VP-glycosides is a challenging task. In addition, purification of crude extract by SPE is usually required to minimize the ion suppression encountered in MS detection [5,11,14]. By contrast, the number of aglycones that are relevant to smoke taint is much smaller and the standards are mostly available. Consequently, we adopted the strategy of measuring the aglycones using GC-MS/MS following a hydrolysis step. 

Various hydrolysis procedures have been reported for glycosylated phenols, and acidic hydrolysis with HCl or H_2_SO_4_ is the most widely used method for this purpose. In the literature related to phenol glycoside hydrolysis for smoke taint analysis, the pH value rather than the acid concentration is frequently given [4,7,14,17]. Given that the cleavage of glycosidic linkages depends on three factors, the acid concentration and incubation temperature and incubation time [24], instead of adjusting the pH of samples, we tried H_2_SO_4_ and HCl at two different concentrations each with a fixed incubation temperature and time; the concentrations were chosen based on published reports for similar purposes [10]. We found that both 1 N HCl and 1.25 N H_2_SO_4_ generate satisfactory and comparable results, whereas a higher concentration of these acids is not advantageous for most bound VPs. Under these hydrolysis conditions, the seven most important smoke taint-related VP compounds appear to be largely stable. However, such a hydrolysis protocol is not suitable for the determination of conjugated isoeugenol; the reason for the very low recovery of isoeugenol in heated acidic medium remains to be determined.

Incomplete hydrolysis of phenol glycosides after a 60-min incubation has been reported by some researchers [14,17]. In this study, we found total disappearance of three model glycosylated VPs upon hydrolysis by 1 N HCl and 1.25 N H_2_SO_4_ for 60 min. In addition, our acid hydrolysis was performed on samples post-LLE (i.e., using aqueous fraction instead of the whole extract) and therefore enabled a clearer differentiation between free and bound VPs. Hexose-guaiacol standard is commercially available, whereas the identity of the other two glycosides is confirmed by their MS2 spectra in comparison to the published data [5]. Procuring more phenol glycoside standards is needed to enable a thorough investigation of the conversion rate under the current hydrolysis conditions. 

A small-scale inter-laboratory comparison revealed that our method generates comparable results as compared to other commercial laboratories that provide a smoke taint analysis service to grape growers. The small difference in VP content observed across different laboratories may result from the different workflows being used. 

When our method was applied to samples having different degrees of bush fire smoke exposure, the level of bound VPs appears to reflect the magnitude of the smoke effect better, while the level of free VPs was low regardless of the sample type. This indicates that measuring bound VPs is necessary for determining the potential risk of producing smoke-tainted wine from smoke-exposed grapes. 

## 4. Materials and Methods

### 4.1. Plant Materials

Red-skinned grapes (Shiraz, Pinot Noir, and Pinot Grigio) and white grapes (Chardonnay) with different levels of bush fire smoke expose (as scored visually by the grape growers) were collected at the post-veraison stage from various vineyards across the state of Victoria, Australia after bush fires that occurred between December 2019 and January 2020. The samples were kept at −20 °C before analysis.

### 4.2. Chemicals and Reagents 

Sixteen VP standards, and one internal standard (IS) *d3*-guaiacol were purchased from Sigma-Aldrich, and one phenol glycoside standard (hexose-guaiacol) from Mcule (Budapest, Hungary) (see Table 1 for the list of the chemicals). Solvents and reagents used for preparing standard calibration solutions, sample extraction, and phenol glycoside hydrolysis included methanol (Merck, Kenilworth, NJ, USA), hexane, ethyl acetate, sulfuric acid (all from Ajax Finechem, Seven Hills, Australia), hydrochloric acid (Scharlau, Barcelona, Spain), and sodium hydroxide (Sigma-Aldrich, St. Louis, MO, USA). 

### 4.3. Extraction of Free and Glycosylated Phenols from Grapes

Two extraction protocols were compared in this work. For protocol 1, 10 g of de-stemmed berries were homogenized in a 50-mL polypropylene falcon tube using a tissue grinder, and then 4 mL of water were added to the homogenate and thoroughly mixed by vortex for 2 min. The samples were centrifuged for 15 min (5000× *g*) and the supernatant was transferred to a new 15-mL tube and the volume adjusted to 10 mL with water. For protocol 2, 2 mL of water and 2 mL of methanol were added to the homogenate, and the samples were incubated in a water bath (80 °C) for 60 min with occasional shaking. After cooling down to room temperature, the samples were centrifuged, and the supernatant adjusted to 10 mL with water.

### 4.4. Sample Preparation for GC-MS

For both free and bound VP measurement, an LLE with hexane/ethyl acetate (1:1, *v*/*v*) as reported by Noestheden et al. [17] was performed prior to GC-MS/MS analysis.

For free VP analysis, 400 µL of hexane/ethyl acetate (1:1, *v*/*v*) containing IS (at 20 ng/mL) were added to 400 µL of the extract and thoroughly mixed for 30 s by vortex. After phase separation, the organic phase (upper phase) was transferred to an injection vial and analyzed directly by GC-MS/MS to quantify the free VPs.

For glycosylated VP analysis, 300 µL of the aqueous phase (lower phase) were hydrolyzed in a heating block (100 °C) for 60 min after adding 300 µL of 2 N HCl, 4 N HCl, 2.5 N H_2_SO_4_, or 5 N H_2_SO_4_ (the final acid concentration in the reaction mixture was 1 N HCl, 2 N HCl, 1.25 N H_2_SO_4_, and 2.5 N H_2_SO_4_, respectively). After cooling to room temperature, the reaction mixture was neutralized with an appropriate amount of 5 N NaOH. After centrifugation (10 min at 13,000× *g*), 200 µL of the hydrolysate were transferred to a new tube and 200 µL of hexane/ethyl acetate (1:1, *v*/*v*) containing IS (at 20 ng/mL) were added to extract the VPs released by hydrolysis. The organic phase was again analyzed directly by GC-MS/MS to determine the conjugated (bound) VPs.

### 4.5. GC-MS/MS Settings

The separation of VPs was achieved using a DB-heavy-wax column (30 m × 0.25 mm ID, 0.25 µm film thickness, Agilent Technologies) with a constant flow of 1.4 mL/min helium as the carrier gas and the following temperature program: Initial temperature of 100 °C and held for 2 min, increased by 10 °C/min to 270 °C, and held for 6 min. The injection inlet temperature was 230 °C and the injection volume was 1 µL in splitless mode.

The detection was by an Agilent 7000 GC/MS Triple Quad with the following settings: Transfer line temperature of 280 °C, source temperature of 250 °C, quad temperature of 150 °C, and a solvent delay of 6 min. An MRM scan mode was employed for the quantification of the 16 VPs and the IS; the detailed MRM parameters are given in Table 1.

### 4.6. LC-MS Analysis of Phenol Glycosides

An RP-LC-MS system was used to verify the hydrolysis efficiency of glycosidically conjugated phenols extracted from grapes with different acids. The glycosylated compounds were separated by an Acquity HSS T3 C18 column (100 × 2.1 mm, 1.8 µm, Waters) on a Vanquish UHPLC system (Thermo Fisher Scientific). The column compartment was maintained at 50 °C and the sample tray at 12 °C. The mobile phase was composed of water containing 0.1% formic acid (A) and acetonitrile containing 0.1% formic acid (B). The gradient elution was performed by a linear increase of mobile phase B from 5% to 50% over 20 min with a flowrate of 0.25 mL/min. The injection volume was 5 µL. 

An Orbitrap Elite mass spectrometer (Thermo Fisher Scientific, Waltham, MA, USA) equipped with a heated electrospray ionization source was used for phenol glycoside detection. The heated capillary was maintained at 275 °C with a source heater temperature of 325 °C, and the sheath, auxiliary, and sweep gases were respectively at 40, 15, and 4 units. The instrument was operated in negative ion mode (3.6 kV) with a full scan (120–1800 *m/z*) at a resolution of 70,000 followed by a data-dependent MS2 scan of targeted parent ions. The raw data acquired were imported into Xcalibur 3.0 software (Thermo Fisher Scientific) for compound identification based on the accurate mass of parent ions as well as MS2 information. 

### 4.7. Method Validation

Determination of the limit of detection (LOD) and the limit of quantitation (LOQ) as well as the linear range for individual VP compounds was carried out using a mixed standard (concentrations ranging from 0.1 to 1000 ng/mL) as described previously [25]. 

To determine the method reproducibility or precision, one sample from each of three grape cultivars (Shiraz, Chardonnay, and Pinot Grigio, all exposed to bush fire smoke) was analyzed five times. The reproducibility for each VP measurement within each cultivar was estimated by the RSD of repeated analyses.

The measurement accuracy of the method was evaluated using the spike recovery test. A mixed standard was spiked (at a level of 100 ng/g) to non-smoked berry homogenate (Shiraz) prior to extraction by the two protocols described above. The recovery was calculated as previously described [25]. In addition, to validate our method, we compared our results with those from three external analytical laboratories (all specialized in smoke taint analysis), using sub-samples collected from the same location. 

### 4.8. Statistical Analysis of Data

For each experiment, the mean values and the standard deviation (SD) or relative standard deviation (RSD) of three technical replicates were presented either in figures or in tables. For statistical comparison between different treatments, the results were subjected to Student’s t-test or ANOVA (XLSTAT, Microsoft Excel); where significant differences (*p* < 0.05) were found between treatments by F test, a Tukey’s honest significant difference (HSD) test was applied for pairwise comparisons. 

## 5. Conclusions

We developed and validated a GC-MS/MS protocol for quantification of both free and conjugated VPs in grape berries. This method involving a small-scale and simple sample preparation procedure for both extraction and hydrolysis is expected to significantly reduce the sample turnover time and analytical cost for the detection of smoke taint in grapes. 

## Figures and Tables

**Table 1:** MRM parameters, LOD, LOQ, and linear range for quantification of 16 phenolic compounds and the internal standard (IS).

Name	Precursor (*m/z*)	Production (*m/z*)	C. E. (eV)	R.T. (min)	LOD (ng/mL)	LOQ (ng/mL)	Linear Range (ng/mL)	*R* ^2^
Guaiacol	124	109	15	11.307	0.33	1	1–1000	0.9998
d3-guaiacol (IS)	127	109	15	11.292	0.33	1	1–1000	0.9998
4-methylguaiacol	138	123	15	12.326	0.33	1	1–1000	0.9998
Syringol	154	139	15	15.320	0.33	1	1–1000	0.9990
4-methylsyringol	168	153	15	16.081	1	3.3	3.3–1000	0.9981
p-cresol	108	107	15	12.712	0.33	1	1–1000	0.9995
o-cresol	108	107	15	13.529	0.33	1	1–1000	0.9996
m-cresol	108	107	15	13.611	0.33	1	1–1000	0.9992
Phenol	94	66	15	12.754	0.33	1	1–1000	0.9993
4-ethylguaiacol	152	137	15	13.101	0.33	1	1–1000	0.9999
Eugenol	164	149	10	14.447	1	3.3	3.3–1000	1.0000
Isoeugenol	164	149	10	16.110	1	3.3	3.3–1000	0.9998
2,4-dimethylphenol	122	107	15	13.501	0.33	1	1–1000	0.9996
4-ethylphenol	122	107	15	14.442	0.33	1	1–1000	0.9996
4-n-propylphenol	136	107	15	15.284	0.33	1	1–1000	0.9997
Vanillin	152	151	15	18.183	1	3.3	3.3–1000	0.9999
Acetovanillone	166	151	15	18.668	1	3.3	3.3–1000	0.9999

**Table 2:** Recovery (% ± SD, *n* = 3) of spiked VPs from berry homogenate with different extraction methods.

VPs	Control	20M
Guaiacol	69.0±1.6 ^b^	81.0±0.3 ^a^
4-methylguaiacol	65.4±2.4 ^b^	77.2±1.2 ^a^
Syringol	71.3±5.0 ^a^	79.6±4.3 ^a^
4-methylsyringol	73.8±3.6 ^a^	76.1±4.2 ^a^
p-cresol	48.3±0.5 ^b^	62.9±2.0 ^a^
o-cresol	63.9±2.2 ^b^	78.6±2.4 ^a^
m-cresol	62.6±2.8 ^b^	75.3±3.2 ^a^
Phenol	55.3±2.5 ^b^	74.2±2.6 ^a^
4-ethylguaiacol	52.8±1.2 ^b^	71.4±3.9 ^a^
Eugenol	59.4±1.3 ^b^	74.0±3.2 ^a^
Isoeugenol	43.9±1.1 ^b^	62.2±5.8 ^a^
2,4-dimethylphenol	46.4±1.1 ^b^	69.7±2.6 ^a^
4-ethylphenol	19.5±0.5 ^b^	51.4±2.0 ^a^
4-*n*-propylphenol	10.9±0.6 ^b^	38.6±2.3 ^a^
Vanillin	54.6±9.8 ^b^	84.9±7.8 ^a^
Acetovanillone	48.5±6.7 ^b^	83.0±4.6 ^a^

Within each compound, recovery values followed by different letters are significantly different (*p* < 0.05).

**Table 3:** Comparison of analytical results across four laboratories.

VPs	VP Form	Lab-A (µg/L) *	Lab-B (µg/g) **	Lab-C (µg/g)	Our Lab (µg/g)
Guaiacol	Free	6.7	9	7	6
	Bound	8.3	3.7	51	16
4-methylguaiacol	Free	<1	1.7	<5	1.1
	Bound	2	-	<5	3.5
Syringol	Free	1	<2	<3	5.1
	Bound	52	37	48	41
4-methylsyringol	Free	<1	<2	<5	< 1
	Bound	9.7	3	8	9.0
o-cresol	Free	4	7.7	<3	3.5
	Bound	<1	-	4	2.7
p-cresol	Free	<1	2	7	1
	Bound	1.3	-	4	5.5
m-cresol	Free	3	6	<3	2.6
	Bound	2	-	8	3.6

* Calculated based on the liquid volume obtained after homogenization of grape berries. ** Bound VPs measured were guaiacol rutinoside, methylguaiacol rutinoside, syringol gentiobioside and methylsyringol gentiobioside for guaiacol, 4-methylguaiacol, syringol, and 4-methylsyringol, respectively.

**Table 4:** Analytical precision (RSD, *n* = 5) for free and bound VPs in three grape cultivars.

VPs	Free VPs						Bound VPs					
	Shiraz		Chardonnay		Pinot		Shiraz		Chardonnay		Pinot	
	Mean	RSD	Mean	RSD	Mean	RSD	Mean	RSD	Mean	RSD	Mean	RSD
	(ng/g)	(%)	(ng/g)	(%)	(ng/g)	(%)	(ng/g)	(%)	(ng/g)	(%)	(ng/g)	(%)
Guaiacol	6.4	7.8	4.4	13.6	7.8	4.1	143.4	5.1	50.7	2.9	41.8	4.1
4-methylguaiacol	1.9	6.8	1.3	17.3	2.4	10.2	37.2	7.9	23.3	7.2	13.2	3.6
Syringol	59.3	8.6	47.7	12.9	40.9	7.2	988.7	11.3	540.7	5.7	394.9	2.2
4-methylsyringol							544.9	11.3	200.7	5.4	147.5	9.0
o-cresol			1.0	11.6	2.6	3.9	13.1	5.9	11.5	5.2	7.7	9.2
p-cresol	1.8	7.4	1.2	10.7	2.8	3.9	16.3	6.4	16.9	3.2	14.1	4.6
m-cresol	1.2	13.7	1.3	8.1	2.9	7.7	17.2	4.0	14.5	7.7	10.8	4.5
Phenol	12.4	5.0	11.9	6.1	17.4	3.7	79.4	4.5	72.4	3.0	61.2	2.0
4-ethylguaiacol							4.3	8.7	4.6	10.3	3.1	8.7
Eugenol							4.0	6.7	5.7	8.9	4.1	21.3
2,4-dimethylphenol							5.9	5.6	6.2	7.8	3.8	10.8
4-ethylphenol	1.4	7.7	1.4	9.8	1.7	9.3	16.7	11.0	10.5	11.2	10.6	10.2
Vanillin	242.4	10.5	206.1	7.8	200.9	3.2	188.0	4.2	120.3	6.7	133.7	2.69
Acetovanillone	9.6	10.5	7.4	8.2	12.9	12.8	74.3	4.7	55.7	3.6	83.6	3.4

Hydrolysis was conducted with 1.25 N H_2_SO_4_ for bound VP determination.

**Table 5:** Free and bound VP content (ng/g) and inter-replicate RSD (in brackets, *n* = 3) of grape berries after exposure to different levels of smoke.

VPs	Free VPs			Bound VPs		
	L	M	H	L	M	H
Guaiacol	2.7 (10.6)	4.3 (4.7)	4.6 (5.2)	24.8 (2.9)	29.3 (6.9)	117.6 (2.8)
4-methylguaiacol	<1	<1	1.5 (6.6)	7.2 (4.8)	12.6 (25.4)	19.3 (3.9)
Syringol	6.4 (15.7)	37.4 (16.4)	17.7 (24.0)	47.1 (20.1)	78.3 (20.9)	486.3 (1.7)
4-methylsyringol	4.5 (9.9)	2.9 (13.6)	6.6 (14.5)	32.2 (18.8)	<2.5	17.0 (19.6)
o-cresol	<1	<1	<1	<2.5	2.5 (16.1)	11.9 (6.1)
p-cresol	<1	<1	<1	6.3 (6.2)	12.5 (7.1)	22.4 (3.9)
m-cresol	<1	<1	<1	4.4 (4.4)	5.6 (5.3)	8.4 (2.4)
Phenol	6.4 (11.2)	6.2 (1.3)	6.6 (6.2)	50.5 (3.7)	88.9 (2.8)	94.7 (12.1)
4-ethylguaiacol	<1	<1	<1	<2.5	<2.5	3.9 (16.7)
Isoeugenol	2.7 (2.7)	3.7 (6.7)	3.2 (32.4)			
2,4-dimethylphenol				<2.5	2.8 (7.6)	6.3 (5.7)
4-ethylphenol	<1	<1	<1	29.8 (5.4)	65.0 (4.4)	30.5 (3.0)
Vanillin	175.6 (4.8)	110.5 (7.6)	219.5 (8.1)	139.7 (4.6)	136.1 (4.9)	160.9 (7.2)
Acetovanillone	5.6 (5.7)	9.7 (7.4)	8.0 (2.8)	43.3 (3.7)	38.4 (5.7)	63.1 (9.3)
Sum of all VPs	204.0	174.7	267.7	385.3	472.0	1042.3

L: low-level smoke; M: medium-level smoke; H: high-level smoke. Hydrolysis was conducted with 1.25 N H_2_SO_4_ for bound VP determination.

## Supplementary Materials

The following are available online at https://www.mdpi.com/2218-1989/10/7/294/s1, Figure S1: LC-MS profile (extracted ion chromatogram) of guaiacol glucoside (A, B, C & D), pentose-hexose-cresol (E, F & G) and guaiacol gentiobioside (H, I & J) before hydrolysis (B, E & H) and after hydrolysis with 1 N HCl (C, F & I) and 1.25 N H_2_SO_4_ (D, G & J). The hydrolysis reaction was conducted at 100 °C for 60 min., Table S1: Recovery of free VP compounds from the heated acidic hydrolysis medium.
