# Supplementary material for: A Simple GC-MS/MS Method for Determination of Smoke Taint-Related Volatile Phenols in Grapes
Source: Metabolites. 2020 Jul 17;10(7):294. doi: 10.3390/metabo10070294 (PMC7407152; doi:10.3390/metabo10070294)
Supplement: Supplementary file 1 [file metabolites-10-00294-s001.pdf]

Supplementary Materials

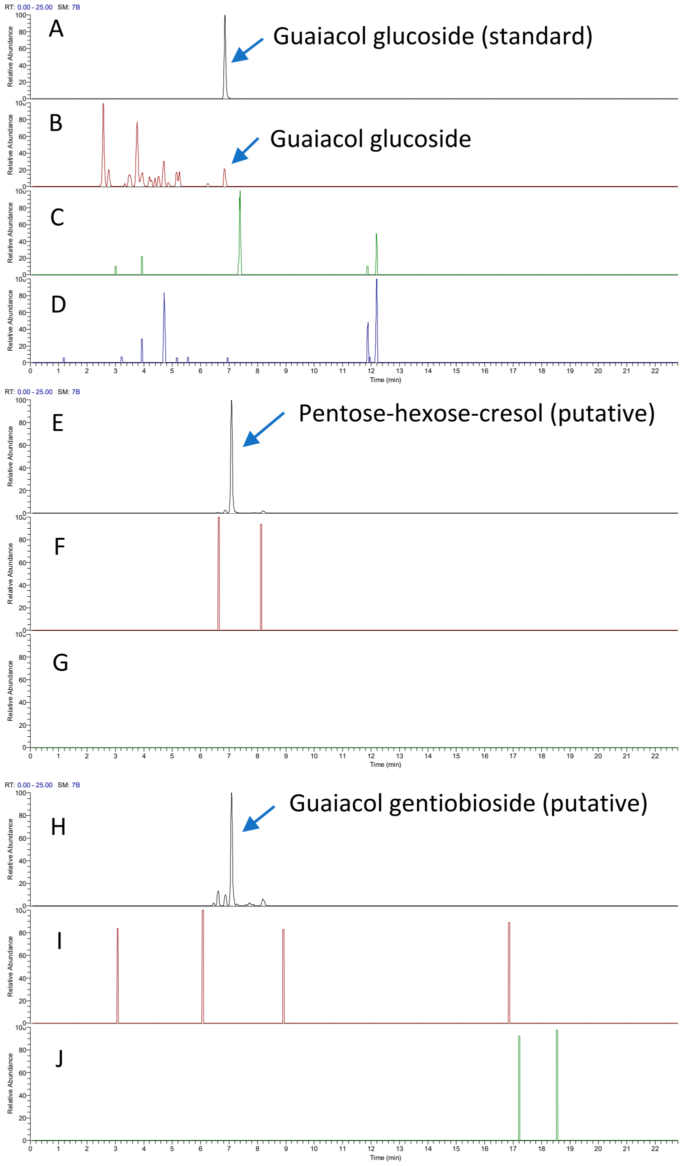

Figure S-1. LC-MS profile (extracted ion chromatogram) of guaiacol glucoside (A, B, C & D), pentose-hexose-cresol (E, F & G) and guaiacol gentiobioside (H, I & J) before hydrolysis (B, E & H) and after hydrolysis with 1 N HCl (C, F & I) and 1.25 N H_2SO_4 (D, G & J). The hydrolysis reaction was conducted at 100 °C for 60 min.

Table S-1: Recovery of free VP compounds from the heated acidic hydrolysis medium

**VPs**	**Recovery (%)**
Guaiacol	87.4±2.8
4-methylguaiacol	81.8±0.2
Syringol	79.4±2.1
4-methylsyringol	80.3±3.9
o-cresol	87.4±2.1
p-cresol	90.5±1.9
m-cresol	80.5±2.6
Phenol	84.9±8.6
4-ethylguaiacol	92.7±6.9
Eugenol	84.1±0.8
Isoeugenol	18.4±4.2
2,4-dimethylphenol	82.7±10.9
4-ethylphenol	84.5±4.0
4-*n*-propylphenol	88.6±13.5
Vanillin	87.4±5.0
Acetovanillone	78.0±8.2
